# Single-port three-dimensional (3D) endoscopic-assisted breast surgery—preliminary results and patient-reported satisfaction in 145 breast cancer and gynecomastia cases

**DOI:** 10.1186/s12957-023-03191-7

**Published:** 2023-10-26

**Authors:** Clement Luck Khng Chia, Chayanee Sae-lim, Hung-Wen Lai, Korawan Chandrachamnong, Hsin-I. Huang, Dar-Ren Chen, Shou-Tung Chen

**Affiliations:** 1 Department of Surgery, Breast Surgery Service, Khoo Teck Puat Hospital, Singapore, Singapore; 2 Department of Surgery, Faculty of Medicine, Chulalongkorn University, Bangkok, Thailand; 3 Department of Surgery, Endoscopic & Oncoplastic Breast Surgery Center, Changhua Christian Hospital, 135 Nanxiao Street, Changhua, 500 Taiwan; 4 Department of Surgery, Division of General Surgery, Changhua Christian Hospital, Changhua, Taiwan; 5 Comprehensive Breast Cancer Center, Changhua Christian Hospital, Changhua, Taiwan; 6 Minimal Invasive Surgery Research Center, Changhua Christian Hospital, Changhua, Taiwan; 7 Kaohsiung Medical University, Kaohsiung, Taiwan; 8 Division of Breast Surgery, Yuanlin Christian Hospital, Yuanlin, Taiwan; 9 School of Medicine, Chung Shan Medical University, Taichung, Taiwan; 10 School of Medicine, National Yang Ming Chiao Tung University, Taipei, Taiwan; 11 Department of Surgery, Division of Breast Surgery, Police General Hospital, Bangkok, Thailand; 12 Department of Information Management, National Sun-Yat-Sen University, Kaohsiung, Taiwan

**Keywords:** Endoscopic-assisted breast surgery (EABS), Single-port 3-dimensional (3D) endoscopic-assisted breast surgery, Nipple-sparing mastectomy (NSM), Breast-conserving surgery (BCS), Breast cancer, Endoscopic gynecomastia surgery, Robotic nipple-sparing mastectomy (R-NSM)

## Abstract

**Background:**

Minimal-accessed (robotic and endoscopic) breast cancer surgery is increasingly performed due to better cosmetic results and acceptable oncological outcomes. This study aims to demonstrate the clinical safety and patient-reported cosmetic satisfaction of single-port three-dimensional endoscopic-assisted breast surgery (S-P 3D EABS), which is our new endoscopic surgical innovation, in both malignant and benign breast conditions.

**Methods:**

Patients who underwent S-P 3D EABS from 1 August 2018 to 31 July 2022 in a single institution were enrolled. Clinical outcomes of this procedure were retrospectively reviewed, and the patient-reported cosmetic satisfaction was evaluated by a questionnaire and reported herein.

**Results:**

During the study period, 145 patients underwent 164 procedures of S-P 3D EABS. One hundred fifty (91.5%) procedures were endoscopic-assisted nipple-sparing mastectomy (S-P 3D E-NSM; 117 therapeutic procedures for breast cancer, 13 prophylactic mastectomies, 20 procedures for gynecomastia). Fourteen (8.5%) procedures of endoscopic-assisted breast-conserving surgery (S-P 3D E-BCS) were performed (12 S-P 3D E-BCS, 2 S-P 3D E-BCS with 3D videoscope-assisted partial breast reconstruction, which was 1 case of latissimus dorsi flap and 1 case of omental flap). The mean operative time was 245 ± 110 min in S-P 3D E-NSM and 260 ± 142 min in S-P 3D E-BCS. The mean intraoperative blood loss was 49.7 ± 46.9 ml in S-P 3D E-NSM and 32.8 ± 17.5 ml in S-P 3D E-BCS. Subnipple biopsy showed positive malignancy in 3 (2.6%) S-P 3D E-NSM patients. None of the S-P 3D E-BCS patients found margin involvement; however, 3 (2.6%) reported margin involvement in S-P 3D E-NSM patients. Thirty-two complications were found (24.6%): 7 (5.3%) transient nipple-areolar complex (NAC) ischemia, 7 (5.3%) partial NAC necrosis, 1 (0.7%) total NAC necrosis, and 1 (0.7%) implant loss. During the mean follow-up time of 34 months, there were 2 (1.5%) patients with locoregional recurrence, 9 (6.9%) distant metastasis, and 2 (1.5%) mortality. 78.6% (77/98) of patients answering the cosmetic-evaluated questionnaire reported good and excellent overall satisfaction.

**Conclusions:**

S-P 3D EABS is a novel surgical innovation, which is able to perform safely in either malignant or benign breast conditions and offer promising cosmetic results.

**Supplementary Information:**

The online version contains supplementary material available at 10.1186/s12957-023-03191-7.

## Background

Currently, the trend towards minimal-accessed breast surgery, endoscopic-assisted breast surgery (EABS), and robotic-assisted breast surgery (RABS) is rising in breast cancer treatment owing to its superior cosmetic results while maintaining oncological outcomes [1–9].

**Fig. 1:**
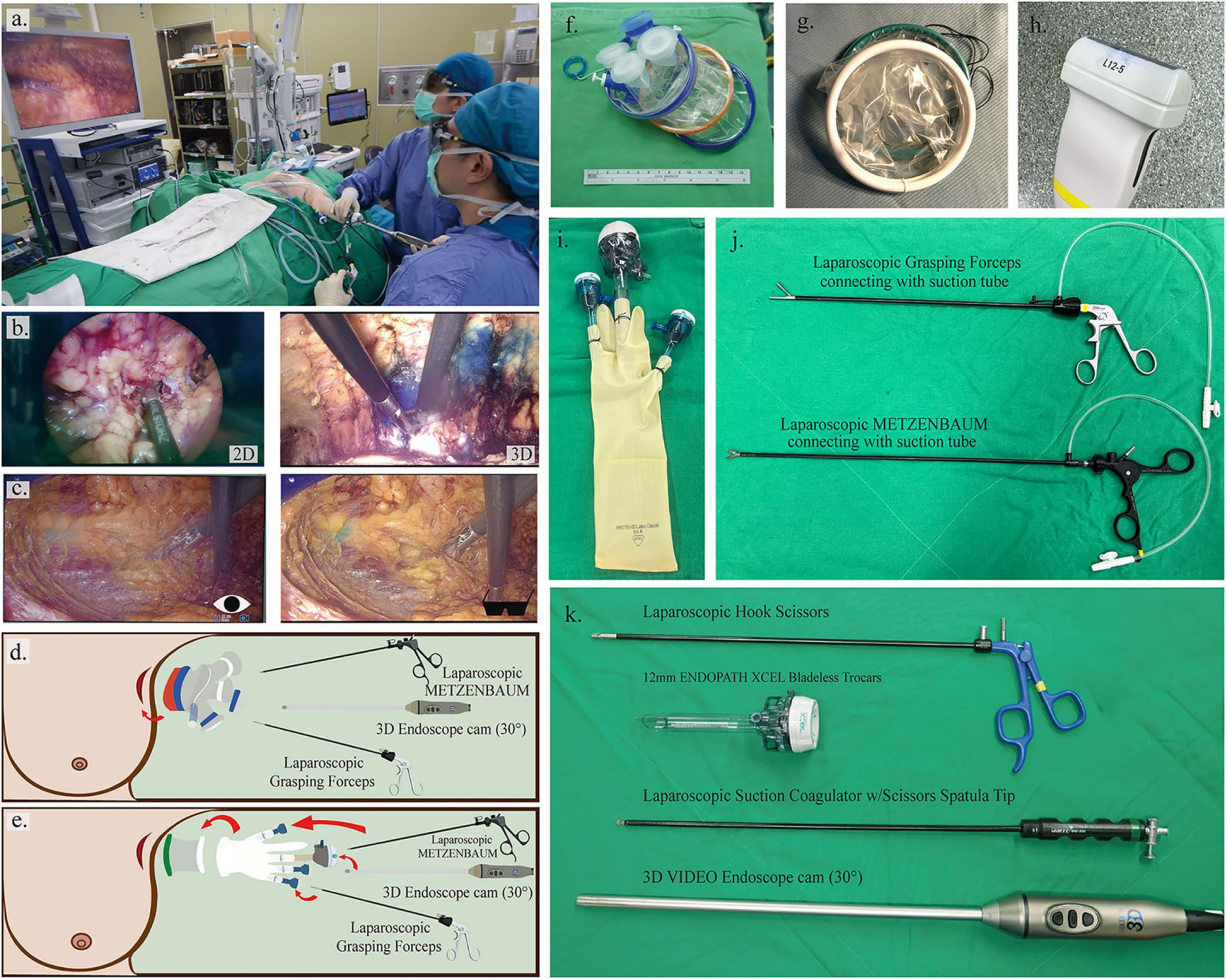
Intraoperative layout and instruments. **a** Intraoperative layout of the single-port three-dimensional endoscopic console with a surgeon and an assistant in the operative field. **b** Operative visualization comparing 2D and 3D system. **c** Visualization of an operative field on the screen comparing with the naked eyes and 3D goggles in the 3D imaging system. **d**, **e** Illustrations demonstrating the insertion of a single port (glove port in **d** and self-made single port in **e**) followed by endoscopic instruments (3D endoscope camera, laparoscopic Metzenbaum scissors, and grasping forceps). **f**–**k** Instruments used in single-port 3D endoscopic breast surgery

We developed the single-port three dimensional (3D) endoscopic-assisted breast surgery (S-P 3D EABS) from our experiences in the dual-incision 2D endoscopic surgery with retractors, which we have done before. First of all, we attempted to perform an endoscopic resection via a single incision in an inconspicuous axillary area, not only to achieve excellent cosmetic outcomes but also to preserve blood supply to the nipple areolar complex (NAC) and skin flap [3]. Therefore, to get an adequate exposure from an incision located distantly from the resected area, an air-insufflation system was utilized instead of a manual retraction. As a result, it could reduce the burdensome of surgical assistance and minimize the risk of skin flap or NAC ischemia/necrosis [3]. Furthermore, we utilized the benefits of the 3D imaging magnification system to enhance surgical precision [3, 10–13](Fig. 1a–c). Lastly, compared with RABS, S-P 3D EABS offers advantages such as reduced cost, fewer instruments required, and the ability to incorporate various oncoplastic techniques [4, 14–18]; therefore, we adapted this technique for both benign and malignant breast surgeries.

This study reports our 4-year experience with S-P 3D EABS, aiming to demonstrate the technique and highlight its clinical safety and patient-report satisfaction in breast cancer and benign breast conditions, including gynecomastia.

## Methods

### Patients

This retrospective study aimed to demonstrate the clinical outcomes and patient-reported satisfaction of our S-P 3D EABS. Patients who underwent S-P 3D EABS from 1 August 2018 to 31 July 2022, in Changhua Christian Hospital (CCH), a tertiary medical center in central Taiwan, were included. The clinicopathologic characteristics collected from the breast cancer database at CCH included age, BMI, tumor location, tumor size, staging, neoadjuvant treatment, types of surgery and reconstruction, histology, molecular subtypes, and adjuvant treatments. All data was collected by specially trained nurses through chart review and confirmed by the principal investigator (HWL) subsequently.

Ethical approval for the study was obtained from the Institutional Review Board of the CCH (CCH IRB No. 141224 and 211228). The current study included photos of several patients who agreed and signed the consent for the publication of their pictures at the outpatient clinic during the follow-up time when we started conducting this study.

### Outcome measures

The main outcomes in our study included perioperative parameters and short-term oncological safety. Perioperative parameters included operative time (separated into time for breast surgery and reconstruction and summarized into total operative time), intraoperative blood loss, length of hospital stay, an incidence of margin and subnipple involvement by malignancy, and complications.

Intra-operative blood loss was measured during the whole operation including breast, axillary surgery, and immediate reconstruction if it was performed. Perioperative hospital stay and breast specimen weight were recorded. Margin involvement by invasive and non-invasive cancer was evaluated in all patients. In all patients who received single-port 3D endoscopic-assisted nipple-sparing mastectomy (S-P 3D E-NSM), subnipple tissue biopsy was performed and sent for intraoperative frozen section and pathological examination. If subnipple biopsy was positive for malignancy, the nipple was excised to perform skin-sparing mastectomy.

Additionally, postoperative complications occurring within 3 months after the surgery were recorded, including delayed wound healing, skin blisters from temporary thermal injury to the skin flap, hematoma and seroma formation, infection, the NAC and skin flap ischemia/necrosis, and implant loss. NAC and skin flap ischemia were evaluated during 2 weeks to 3 months postoperatively. The eventual survival of NAC and skin flap was confirmed at 3 months of follow-up visit. The viability of NAC was graded into transient ischemia (recovering without loss of nipple volume), partial necrosis (recovering with partial loss of nipple volume), and total necrosis (leading to total loss of nipple volume) [3]. The severity of skin flap necrosis was graded as temporal color change (cyanosis or erythema), partial thickness skin necrosis (resulting in at least epidermal sloughing), and full-thickness skin flap necrosis [19, 20]. Additionally, we evaluated the extent of the involved surface area by eyeball assessment in the outpatient clinic and categorized it into 1–10%, 11–30%, and > 30% of the entire breast area [19, 20]. Implant loss was defined as an explantation due to surgery-related complications within 3 months of reconstruction [21]. Complication severity was assessed and reported as the Clavien-Dindo (CD) classification [22].

Furthermore, oncologic safety was evaluated through the incidence of locoregional and distant recurrence and breast cancer-specific mortality after S-P 3D EABS. Locoregional recurrence was defined as cancer reappearance at the operative site [23], and distant recurrence was defined as any recurrence in distal organs [23]. Incidence of recurrence and mortality due to breast cancer was ascertained at the most recent follow-up, which ended on 31 August 2023.

### Evaluation of postoperative aesthetic outcomes

Patient-reported cosmetic results were evaluated in an outpatient clinic 3 months after the operations using a questionnaire, which we developed to survey aesthetic outcomes following minimal-accessed breast surgery in our center [3–5, 7, 24, 25]. A questionnaire consisted of 10 questions, regarding the satisfaction with the scar (appearance, length, and position), overall cosmetic results with clothing, satisfaction in size and symmetry compared with the other side, satisfaction with NAC position, and willingness to choose S-P 3D E-NSM again if they have a second chance to do so. In each question, patients scored 1 (poor), 2 (fair), 3 (good), and 4 (excellent). An overall satisfaction was defined as a summary of scores from questions 2 to 9, which was categorized into excellent (total scores of 28–32), good (total scores of 20–27), fair (total scores of 12–19), and poor (total scores of 8–11). Patients with aesthetic results of “excellent” or “good” are defined as satisfied with the cosmetic outcome. The contents of our questionnaire are demonstrated in Table 4.

### Indications of single-port 3D endoscopic-assisted breast surgery (S-P 3D EABS)

Patients were evaluated an eligibility for S-P 3D EABS using preoperative breast sonography, mammography, and/or breast magnetic resonance imaging (MRI). Additionally, liver sonography, chest X-ray, and whole-body bone scan were used to exclude the possibility of distant metastasis. The indications of S-P 3D E-NSM [3, 11] included early-stage breast cancer (ductal carcinoma in situ (DCIS), stage I, II, or IIIA), a tumor size ≤ 5 cm, no apparent multiple lymph node metastases, and no evidence of nipple, skin, or chest wall invasion [3, 23, 25]. S-P 3D E-NSM was contraindicated in any patients with apparent NAC involvement, inflammatory breast cancer, breast cancer with chest wall or skin invasion, locally advanced breast cancer, breast cancer with extensive axillary lymph node metastasis (stage IIIB or later) [3, 23, 25].

Indications for single-port 3D endoscopic-assisted breast-conserving surgery (S-P 3D E-BCS) [7] included early-stage breast cancer (ductal carcinoma in situ (DCIS), stage I or II cancer), tumor size < 3 cm, no evidence of multiple lymph nodes metastasis, and no evidence of skin or chest wall invasion. Patients for whom S-P 3D E-BCS were contraindicated included those with inflammatory breast cancer, multicentric disease, diffuse suspicious or malignant microcalcifications, breast cancer with chest wall or skin invasion, locally advanced breast cancer, breast cancer with extensive axillary lymph nodes metastasis (stage IIIB and above), and contraindicated to radiotherapy [26]. Multifocal disease was not contraindicated when all the lesions could be removed en bloc in a single excision.

In addition, patients with severe comorbidity, such as heart disease, renal failure, liver dysfunction, and poor performance status as assessed by the primary physicians, were not suitable as good candidates for S-P 3D EABS [3, 23, 25].

### Surgical procedures

**Fig. 2:**
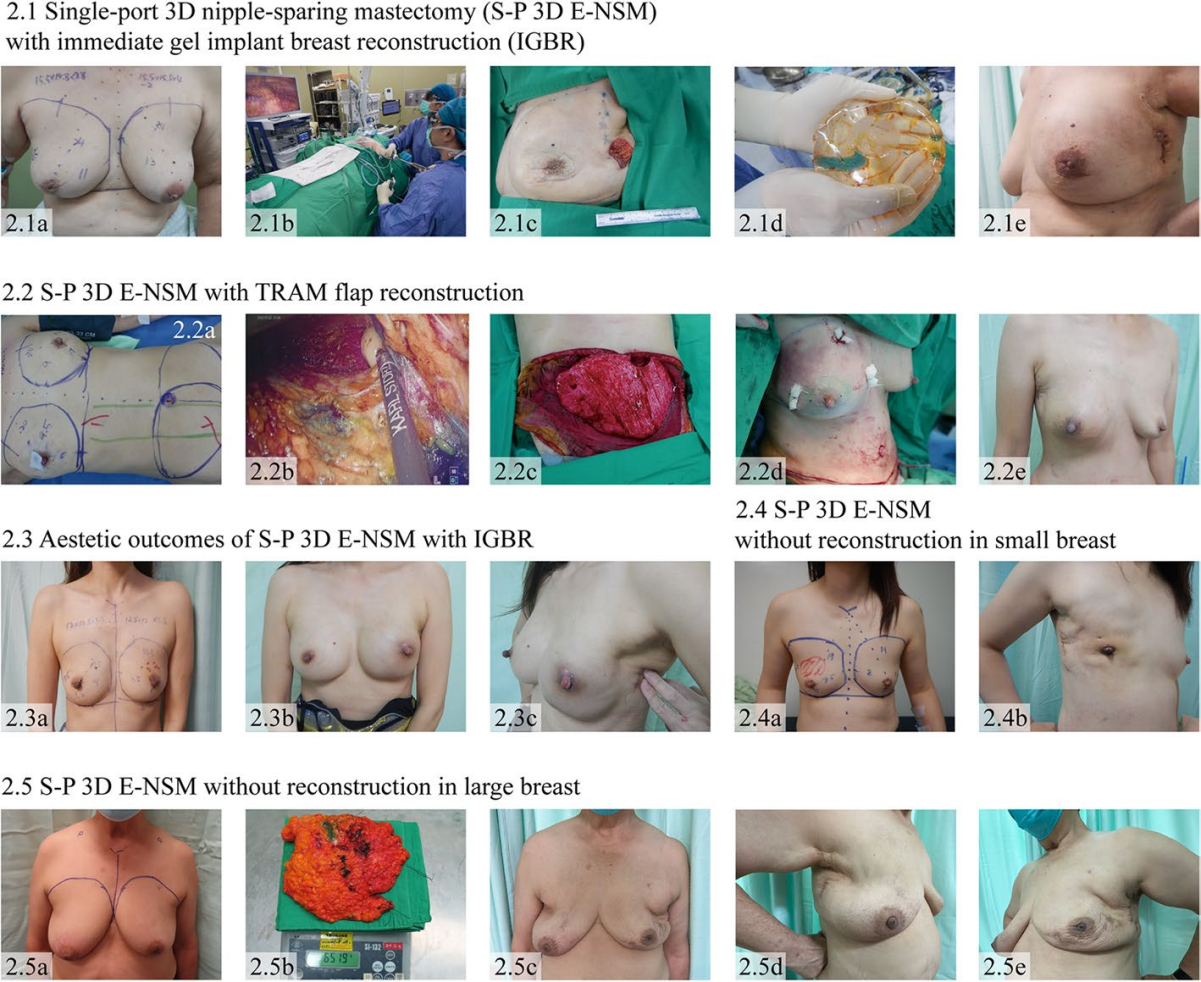
Single-port 3D endoscopic-assisted nipple-sparing mastectomy (S-P 3D E-NSM). 2.1 S-P 3D E-NSM with immediate gel implant breast reconstruction (IGBR) for left breast cancer. (2.1a) Preoperative marking. (2.1b) Intraoperative layout. (2.1c) Immediate postmastectomy appearance showing a small incision placed inconspicuously in the axilla. (2.1d) Cohesive gel implant used for breast reconstruction. (2.1e) Postoperative appearance after S-P 3D E-NSM with IGBR. 2.2 S-P 3D E-NSM with transverse rectus abdominis myocutaneous (TRAM) flap reconstruction for right breast cancer. (2.2a) Preoperative marking performed before general anesthesia. (2.2b) Endoscopic dissection of breast skin flap performed with monopolar laparoscopic Metzenbaum scissors. (2.2c) Intraoperative view of a pedicle TRAM flap. (2.2d) Immediate postoperative view after TRAM flap reconstruction. (2.2e) Aesthetic outcomes at 2 months after S-P 3D E-NSM with TRAM flap reconstruction. 2.3 Aesthetic result in S-P 3D E-NSM with IGBR for left breast cancer. (2.3a) Preoperative marking. (2.3b) Anterior view of postoperative appearance after S-P 3D E-NSM with IGBR. (2.3c) Lateral view of postoperative appearance after S-P 3D E-NSM with IGBR. 2.4 Aesthetic result after S-P 3D E-NSM without reconstruction for right breast cancer in a patient with small breasts. (2.4a) Preoperative marking. (2.4b) Lateral view of postoperative appearance after S-P 3D E-NSM without reconstruction. 2.5 Aesthetic result in S-P 3D E-NSM without reconstruction for bilateral breast cancer in a patient with large breasts. (2.5a) Preoperative marking. (2.5b) Mastectomy specimen weight 652 g, right side. (2.5c) Anterior view of postoperative appearance after S-P 3D E-NSM without reconstruction. (2.5d, 2.5e) Lateral view of postoperative appearance after S-P 3D E-NSM without reconstruction

**Fig. 3:**
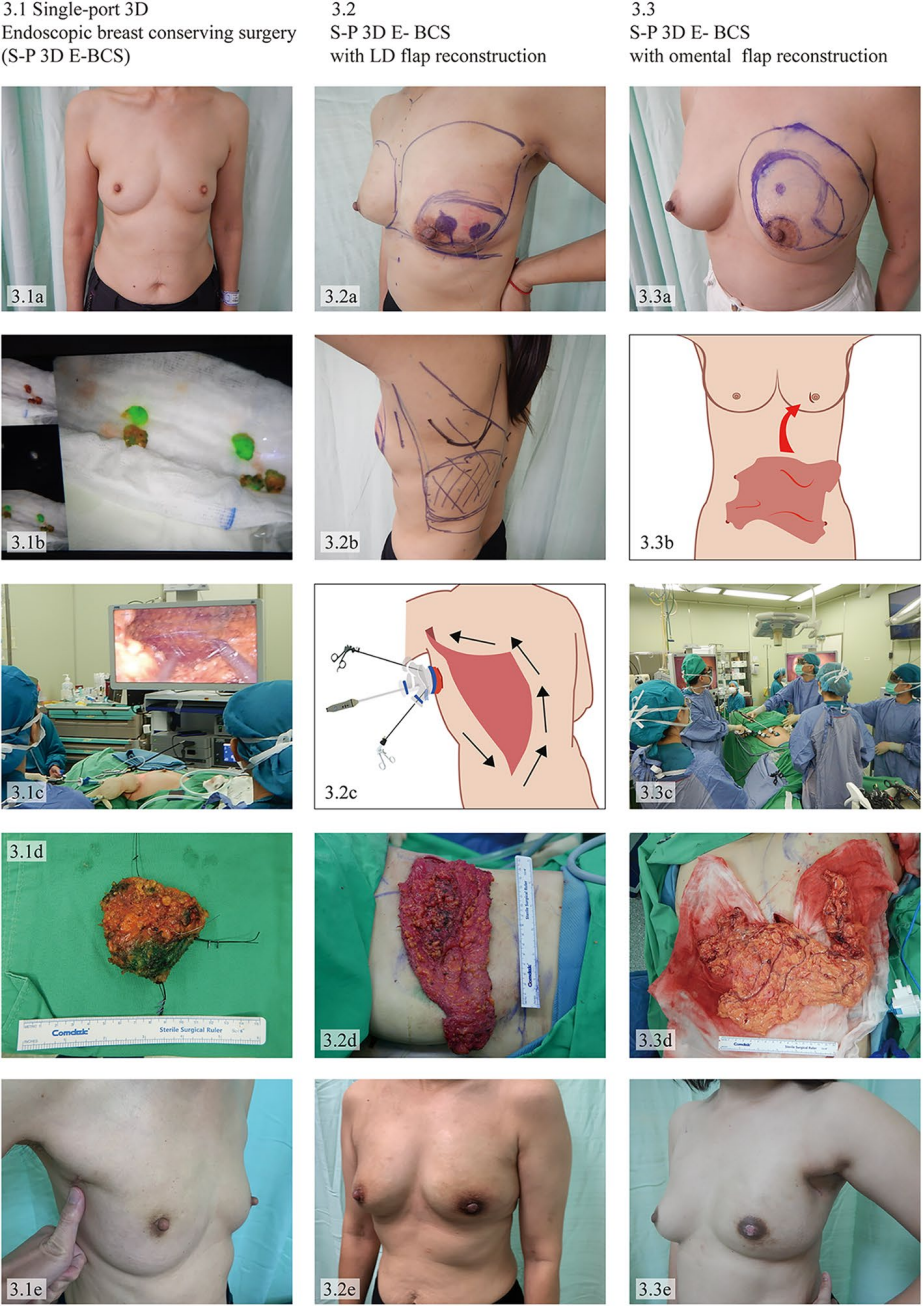
Single-port 3D endoscopic-assisted breast-conserving surgery (S-P 3D E-BCS). 3.1 S-P 3D E-BCS for right breast cancer. (3.1a) Preoperative appearance. (3.1b) Sentinel lymph node biopsy identified by ICG & Technetium-99m and retrieved via the same incision as S-P 3D E-BCS. (3.1c) Intraoperative layout. (3.1d) The breast specimen which was removed through the wound and oriented for routine histopathologic examination. (3.1e) A single small incision for S-P 3D E-BCS at inconspicuous area. 3.2 S-P 3D E-BCS with 3D videoscope-assisted latissimus dorsi (LD) flap reconstruction for left breast cancer. (3.2a, 3.2b) Preoperative marking. (3.2c) Illustrations demonstrating the insertion of a single port for harvested LD flap. (3.2d) Intraoperative view of an LD flap. (3.2e) Postoperative appearance after S-P 3D E-BCS with LD flap reconstruction. 3.3 S-P 3D E-BCS with 3D videoscope-assisted harvest of omental flap reconstruction for left breast cancer. (3.3a) Pre-operative marking. (3.3b) Illustrations demonstrating the incision at an intra-mammary fold for S-P 3D E-BCS with 3D videoscope-assisted harvest of omental flap reconstruction. (3.3c) Intraoperative layout for 3D videoscope-assisted harvest of the omental flap. (3.3d) Intraoperative view of an omental flap. (3.3e) Postoperative appearance after S-P 3D E-BCS with 3D videoscope-assisted harvest of omental flap reconstruction

**Fig. 4:**
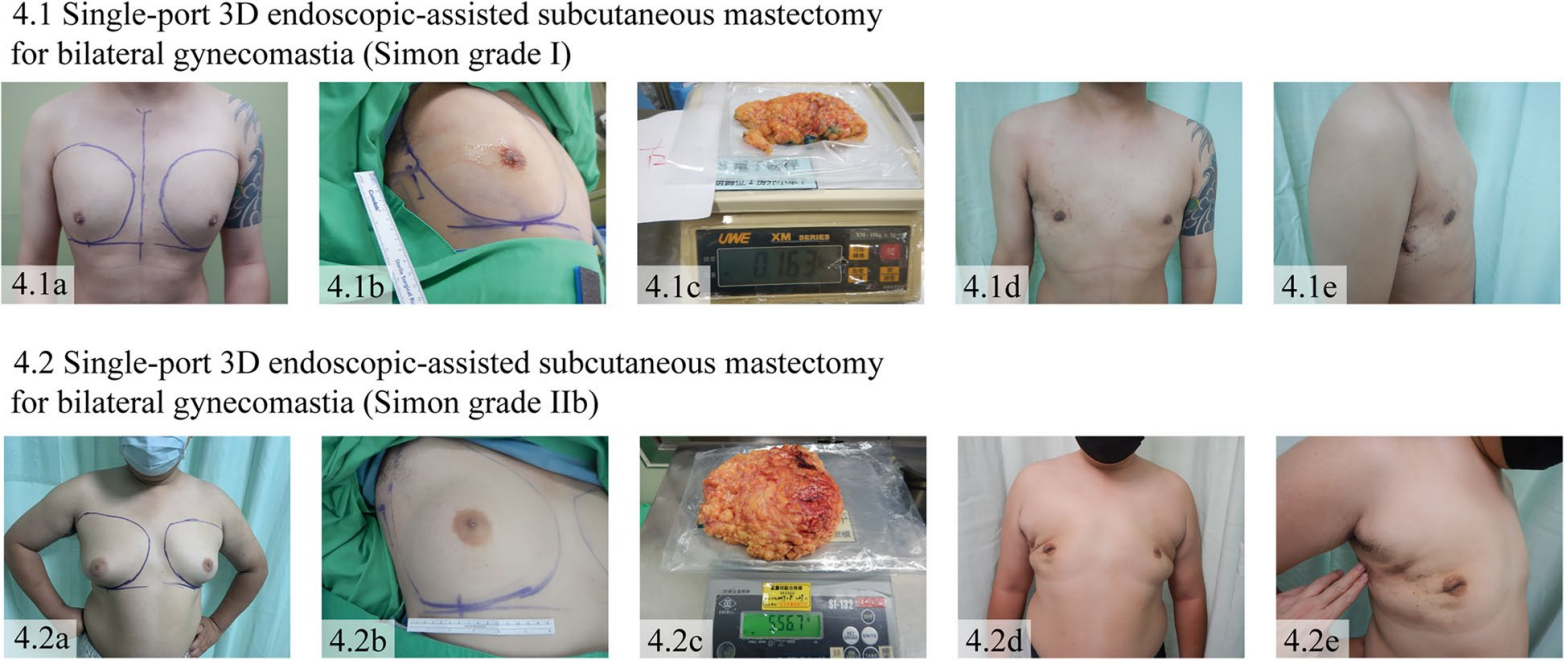
Single-port 3D endoscopic-assisted (S-P 3D E-) subcutaneous mastectomy for bilateral gynecomastia. 4.1 S-P 3D E-subcutaneous mastectomy in Simon grade I. (4.1a) Preoperative appearance. (4.1b) Pre-operative marking. (4.1c) Breast specimen that was removed via an incision at lateral chest and the specimen weight 163 g. (4.1d, 4.1e) Anterior and lateral view of postoperative appearance. 4.2 S-P 3D E-subcutaneous mastectomy in Simon grade IIb. (4.2a) Preoperative appearance. (4.2b) Preoperative marking. (4.2c) Breast specimen that was removed via an incision at lateral chest and the specimen weight 556.7 g. (4.2d, 4.2e) Anterior and lateral view of postoperative appearance

The surgical techniques of S-P 3D E-NSM for breast cancer [3, 11] and S-P 3D E-subcutaneous mastectomy for gynecomastia [27] have been previously described. Preoperative marking was performed in the standing position. After anesthesia induction, the patient was aligned in a supine position with the ipsilateral arm abducted at 90°. The ipsilateral shoulder was then elevated to 30° to facilitate access to an axilla [11]. The location and length of the skin incision varied depending on the specific indication (Figs. 1, 2, 3, and 4). In S-P 3D E-NSM (Figs. 1 and 2) or S-P 3D E-BCS (Fig. 3), the incision could be made in the axilla area to facilitate axillary lymph node surgery, either sentinel lymph node biopsy (SLNB) or axillary lymph node dissection (ALND). In gynecomastia (Fig. 4), the incision was placed at the anterior axillary line at the level of NAC. The length of the skin incision ranged from 2.5 to 5 cm depending on the size of the specimen and the disease nature. In cases with large breasts, the incision size could be extended up to 6 cm to facilitate specimen removal.

In breast cancer cases, conventional SLNB was performed via an axillary incision with the standard dual tracers, using indocyanine green and colloidal human serum albumin labeled with technetium-99 m (Tc-99 m) before the 3D EABS. After sentinel nodes were retrieved, we sent them for frozen section analysis. If macrometastasis was found in the frozen section meeting the criteria for ALND, a conventional ALND was continued in the same operation via the same incision or extending the axillary incision for 1–2 cm if it is necessary to obtain better exposure under a lighted retractor.

For S-P 3D E-NSM, methylene blue mixed with xylocaine jelly was injected from the skin to the retroglandular fat to mark the boundary of the breast for guidance during endoscopic dissection [11, 15, 24, 25]. Hydro-dissection was performed by injecting a tumescent solution containing lactated Ringer’s solution, 0.05% lidocaine, and epinephrine 1:1,000,000 into subcutaneous tissue from the NAC to the peripheral boundary of the breast. A subcutaneous skin flap is first dissected under direct vision for 2–3 cm from an axillary incision to create a working space for the placement of the single port (Glove Port; Nelis, Gyeonggi-do, Korea (Fig. 1d, f) or a glove-made single port (Fig. 1e, g, i). After port placement, carbon dioxide insufflation at a pressure of 8–10 mmHg was then performed to create space [3, 11]. A 30° 10-mm diameter camera TIPCAM 1 S 3D VIDEO Endoscope (KARL STORZ, Germany) was inserted via a 12-mm trocar (Fig. 1d, e, k). For an optimal 3D visualization, a 30° upward angle with reverse 180° imaging was used during the dissection of the outer part of the breast, then turn to a 30° downward angle while dissection of the inner part of the breast.

The dissection was carried out using laparoscopic diathermy curved Metzenbaum scissors (KARL STORZ, Germany) and counteracted by laparoscopic grasping forceps, which were connected to Nelaton suction tubes no.12 to evacuate the smoke created during the surgery (Fig. 1j). To utilize the lifting benefit of an air inflation system, posterior (retroglandular) dissection was performed first, followed by the anterior (subcutaneous) dissection by creating subcutaneous tunnels using Metzenbaum scissors to facilitate and guide the direction of the endoscopic subcutaneous dissection.

To avoid thermal burns to the NAC, laparoscopic hook scissors (Snowden Pencer; BD, USA, Fig. 1k) were used to sharply cut the dense ductal tissue under the NAC. Subnipple tissue was sent for the frozen section analysis in all breast cancer patients who underwent S-P 3D E-NSM by taking two separate specimens (the inner part from coring out the nipple and the outer part from the breast specimen). If a malignant invasion was found in the subnipple area, the entire NAC was removed to perform skin-sparing mastectomy (SSM).

After mastectomy, immediate breast reconstruction was performed if indicated. An option for breast reconstruction, including implant-based or autologous flap procedures, is based on shared decision-making between the patient and surgeon. We performed immediate subpectoral gel implant-based breast reconstruction (IGBR) (Fig. 2(2.1, 2.3)), by lifting the pectoralis major muscle using laparoscopic grasping forceps and dissecting the plane with laparoscopic Metzenbaum scissors or spatula tip suction coagulator. Then, cohesive gel implant was inserted via the axillary incision into a subpectoral muscular pocket formed by pectoralis major, serratus anterior, and fascia of the external oblique muscle [3, 11, 28]. The autologous flaps, such as the pedicle transverse rectus abdominis myocutaneous (TRAM) flap [14] (Fig. 2(2.2)), were placed in the pre-pectoral plane. Before wound closure with absorbable sutures (Vicryl 3–0 and Monocryl 4–0), 2 closed suction drains were placed, which were one in the subpectoral pocket and another above the pectoralis muscle. As for the patients receiving S-P 3D E-NSM “without” reconstruction, the skin was retracted gradually after the removal of the breast tissue leaving a small single incision and an overall breast-like appearance without any skin reduction procedure (Fig. 2(2.4, 2.5)).

In S-P 3D E-BCS, intraoperative ultrasonography with a linear probe 50 mm, 12 MHz (Fig. 1h), was used to locate the tumor site, and the resection margin was marked using methylene blue mixed with xylocaine gel at 1.5 cm beyond the tumor border. Xylocaine gel helped contain the blue dye in place; as a result, it could prevent an over-resection. [7, 29]. Contrary to S-P 3D E-NSM, the endoscopic dissection in S-P 3D E-BCS began with an anterior (subcutaneous) dissection from an axillary incision to access the tumor resection margin, followed by the parenchymal transection to expose the pectoral fascia using the same instruments as in S-P 3D E-NSM (laparoscopic metzenbaum scissors and monopolar Endo hook). Then, the posterior dissection (retroglandular) dissection was continued, and circumferential parenchymal transection was then carried out to access all the blue-marked resection margin. The entire breast specimen was removed through an axillary incision.

After specimen removal, intraoperative margin assessments were performed with intraoperative specimen ultrasound and mammography. Oncoplastic level I repair with volume displacement technique was performed by laparoscopic suturing of the glandular tissue to minimize postoperative defects (Fig. 3(3.1)). No close-suctioned drain was required in S-P 3D E-BCS without reconstruction. For larger defects, volume replacement using 3D videoscope-assisted partial breast reconstruction techniques was considered [16–18], for example, latissimus dorsi myocutaneous (LD) flap (Fig. 3(3.2)) or omental flap (Fig. 3(3.3)). Then, a close-suctioned drain was placed before the wound closure.

### Statistical analyses

Differences in continuous variables were tested by the independent *t*-test and reported as means ± standard deviations. The chi-square test was used for categorical comparisons of data when appropriate. A *P*-value lower than 0.05 indicates statistical significance. All tests were two-tailed, and all statistical analyses were performed with the statistical package SPSS (version 22.0, SPSS, Chicago, IL, USA).

## Results

### Patient characteristics

A total of 145 patients with 164 breast operations of 3D EABS were recruited at CCH. 117 (71.3%) procedures of single-port 3D for E-NSM, and 14 (8.5%) procedures of S-P 3D E-BCS were performed for breast cancer treatment. There were 13 (7.9%) procedures of S-P 3D E-prophylaxis mastectomy (9 contralateral risk reduction performed in the same operation with therapeutic S-P 3D E-NSM in breast cancer patients, 4 bilateral prophylactic mastectomies in 2 high-risk patients). After this technique was developed in breast cancer treatment, we applied the concept of S-P 3D EABS to S-P 3D E-subcutaneous mastectomy for patients with gynecomastia (10 patients with bilateral subcutaneous mastectomies (20 procedures, 12.2%) (Fig. 4).

Among 117 breast cancer patients who underwent S-P 3D E-NSM, the mean age of the patients was 52 ± 10.8 years. 28.2% (35/117) of patients received neoadjuvant chemotherapy. Breast reconstructions were performed in 66.7% (78/117) of procedures (71 gel implant reconstruction, 7 TRAM flap). S-P 3D E-NSM without reconstruction was performed in 33.3% (39/117) of them (Fig. 2). The mean pathologic tumor size was 2.9 ± 2.5 cm. Lymph node metastasis was presented in 39.9% (35/117) of them. 87.2% of the therapeutic S-P 3D E-NSM patients had pathologic stages 0–II (30.8% stage 0/pCR, 17.9% stage I, 38.5% stage II).

From 14 cases receiving S-P 3D E-BCS, the mean age of patients was 55.8 ± 9.9 years. 14.3% (2/14) of them received neoadjuvant chemotherapy. The mean pathologic tumor size was 2.1 ± 1.1, cm and lymph node metastasis was found in 14.3% (2/14). All patients who undergone S-P 3D E-BCS had pathological stages 0–II. 3D videoscope-assisted partial breast reconstructions were performed in 2 cases (1 case of LD flap and 1 case of omental flap reconstruction) (Fig. 3). Characteristics of breast cancer patients who underwent S-P 3D EABS were summarized in Table 1.

**Table 1:** Characteristics of cancer patients who received therapeutic single-port 3-dimensional endoscopic-assisted breast surgery (S-P 3D EABS)

	**ALL (*N* = 131)**	**S-P 3D E-NSM (*N* = 117)**	**S-P 3D E**-**BCS** **(*N* = 14)**
**Age, years, *N* (%)**			
** < 40**	13 (9.9)	13 (11.1)	0
**≧ 40, < 60**	81 (61.8)	72 (61.5)	9 (64.3)
**≧ 60**	37 (28.2)	32 (27.3)	5 (35.7)
**Mean ± SD**	53.2 ± 10.7	52 ± 10.8	55.8 ± 9.9
**BMI (kg/m**^**2**^**), *N* (%)**			
** < 18**	2 (1.6)	2 (1.7)	0
**18–24**	70 (53.4)	62 (53)	8 (57.1)
** > 24**	59 (45)	53 (45.3)	6 (42.9)
**Location, *N* (%)**			
**Right**	74 (56.5)	66 (56.4)	8 (57.1)
**Left**	57 (43.5)	51 (43.6)	6 (42.9)
**Sonographic tumor size, cm, mean ± SD**	2.5 ± 1.2	2.6 ± 1.2	2.2 ± 1
**Neoadjuvant, *N* (%)**			
**Yes**	35 (26.7)	33 (28.2)	2 (14.3)
**No**	96 (73.3)	84 (71.8)	12 (85.7)
**Axillary surgery, *N* (%) (NA = 4)**			
**SLNB**	100 (78.7)	87 (77)	13 (92.9)
**SLNB + ALND**	25 (19.7)	24 (21.2)	1 (7.1)
**ALND**	2 (1.6)	2 (1.8)	0
**Reconstruction, *N* (%)**			
**No reconstruction**	51 (38.9)	39 (33.3)	12 (85.8)
**Gel implant**	71 (54.2)	71 (60.7)	0
**TRAM flap**	7 (5.3)	7 (6)	0
**LD flap**	1 (0.8)	0	1 (7.1)
**Omental flap**	1 (0.8)	0	1 (7.1)
**Pathologic tumor size, cm, mean ± SD**	2.7 ± 2.4	2.9 ± 2.5	2.1 ± 1.1
**Histology, *N* (%)**			
**DCIS**	33 (25.2)	32 (27.4)	1 (7.1)
**IDC**	79 (60.3)	68 (58.1)	11 (78.6)
**ILC**	8 (6.1)	7 (6)	1 (7.1)
**Others**^**a**^	11 (8.4)	10 (8.5)	1 (7.1)
**Pathological *N* status, *N* (%)**			
**N0**	94 (71.8)	82 (70.1)	12 (85.7)
**N1**	27 (20.6)	25 (21.4)	2 (14.3)
**N2**	7 (5.3)	7 (6)	0
**N3**	3 (2.3)	3 (2.5)	0
**Pathological staging, *N* (%)**			
**0/pCR**	38 (29)	36 (30.8)	2 (14.3)
**I**	28 (21.4)	21 (17.9)	7 (50)
**II**	50 (38.2)	45 (38.5)	5 (35.7)
**III**	15 (11.4)	15 (12.8)	0
**ER, *N* (%) (NA = 12)**			
**Positive**	95 (79.8)	84 (79.2)	11 (84.6)
**Negative**	24 (20.2)	22 (20.8)	2 (15.4)
**PR, *N* (%) (NA = 14)**			
**Positive**	75 (64.1)	67 (64.4)	8 (61.5)
**Negative**	42 (35.9)	37 (35.6)	5 (38.5)
**HER-2, *N* (%) (NA = 30)**			
**Positive**	18 (17.8)	16 (18.2)	2 (15.4)
**Negative**	83 (82.2)	72 (81.8)	11 (84.5)
**Ki-67, *N* (%) (NA = 40)**			
**≦ 14**	41 (45)	35 (44.9)	6 (46.2)
** > 14**	50 (55)	43 (55.1)	7 (53.8)
**Endocrine therapy, *N* (%) (yes)**	86 (65.7)	76 (65)	10 (71.4)
**Chemotherapy, *N* (%) (yes)**	63 (48.1)	58 (49.6)	5 (35.7)
**Radiotherapy, *N* (%) (yes)**	50 (38.2)	38 (32.5)	12 (85.7)

*S-P 3D E-NSM* single-port 3-dimensional endoscopic-assisted nipple-sparing mastectomy, *S-P 3D E-BCS* single-port 3-dimensional endoscopic-assisted breast-conserving surgery, *BMI* body mass index, *ALN* axillary lymph node, *SLNB* sentinel lymph node biopsy, *ALND* axillary lymph node dissection, *TRAM flap* transverse rectus abdominis myocutaneous flap, *LD flap* latissimus dorsi myocutaneous flap, *SD* standard deviation

^a^Other pathologies included solid papillary carcinoma, mucinous carcinoma, neuroendocrine tumor, and no residual tumor in pathological complete response (pCR)

### Perioperative outcomes

Among 117 S-P 3D E-NSM procedures for breast cancer patients, the mean total operative time was 245 ± 110 min (187 ± 83 min for S-P 3D E-NSM without reconstruction, 242 ± 84 min for S-P 3D E-NSM with gel implant reconstruction, 545 ± 100 min for S-P 3D E-NSM with TRAM flap). The mean blood loss was 49.7 ± 46.9 ml. Intraoperative subnipple biopsy was performed in all patients, with 2.6% (3/117) of the biopsies yielding positive results and requiring conversion to skin-sparing mastectomy. The mean hospital stay was 6 ± 3 days. Margin involvement was found in 3 (2.6%, 3/117) patients who received S-P 3D E-NSM. Partial NAC ischemia occurred in 7 patients (6%, 7/117), and total NAC necrosis was found in 1 patient (0.8%, 1/117). Temporal color change from decreased blood flow on 1–10% of skin flap area was observed in 4 patients (3.4%, 4/117). None of them suffered from full- or partial-thickness skin flap necrosis. In addition, 95% (18/19) of all skin and NAC ischemic events have been successfully treated with local wound care with neomycin ointment (CD I) [22], except for 1 patient with total NAC necrosis was treated with local debridement at the outpatient clinic (CD IIIa) [22]. Implant loss was reported in 1 patient who experienced an implant infection (0.8%, 1/117).

For the 14 cases of S-P 3D E-BCS, the mean blood loss was 32.8 ± 17.5 ml, and the mean operative time was 260 ± 142 min (233 ± 92 min for S-P 3D E-BCS without reconstruction). The mean specimen weight was 76.9 ± 41.4 g. The length of hospital stay was 3.9 ± 0.8 days. No resection margin involvement or major complications were reported. Only one patient in this group reported seroma, categorized in CD classification I.

Regarding oncological safety, during the mean follow-up duration of 34.5 ± 15.6 (10–60) months, local recurrences were observed in 1.5% (2/131) of cases, distant metastasis in 6.9% (9/131) of cases, and two mortality (1.5%) was reported from the disease progression in patients who initially diagnosed as locally advance breast cancers. Perioperative and oncological outcomes of therapeutic S-P 3D EABS in breast cancer patients were demonstrated in Table 2.

**Table 2:** Perioperative parameters in cancer patients who undergone therapeutic single-port 3-dimensional endoscopic-assisted breast surgery (S-P 3D EABS)

	**ALL** **(*N* = 131)**	**S-P 3D E-NSM (*n* = 117)**	**S-P 3D E**-**BCS (*n* = 14)**
**Operative time, min, mean ± SD (median, range)**			
**Total time**	247 ± 113 (212, 85–720)	245 ± 110 (210, 92–720)	260 ± 142 (227, 85–615)
**Breast surgery without reconstruction**	191 ± 84 (169, 65–450)	187 ± 83 (164, 65–420)	233 ± 92 (227, 85–450)
**Breast surgery with gel implant reconstruction**	–	242 ± 84 (222, 118–494)	–
**Breast surgery with autologous reconstruction**	–	548 ± 100 (524, 430–720)	**Omental** 615 **LD** 485
**Reconstruction time, min, mean ± SD (median, range)**			
**Gel implant reconstruction**	–	72 ± 24 (60.5, 35–178)	–
**Autologous reconstruction**	–	221 ± 36 (210, 190–300)	**Omental** 165 **LD** 210
**Blood loss, ml, mean ± SD (median, range)**	48.4 ± 45.1 (40, 20–260)	49.7 ± 46.9 (40, 20–260)	32.8 ± 17.5 (25, 20–80)
**Conversion to open surgery,** ***N*** **(%)**	0	0	0
**Length of hospital stay, days, mean ± SD (median, range)**	5.8 ± 2.9 (5, 1–28)	6 ± 3 (6, 1–28)	3.9 ± 0.8 (4, 3–6)
**Breast specimen weight, g, mean ± SD (median, range)**	322.9 ± 177.3 (307, 33–850)	349.4 ± 165.5 (313.5, 36–850)	76.9 ± 41.4 (67, 33–193)
**Subnipple tissue biopsy,** ***N*** **(%) (NA = 3)**			
**Positive**	–	3 (2.6)	–
**Negative**	–	111 (97.4)	–
**Margin status,** ***N*** **(%)**			
**Involved**	3 (2.3)	3 (2.6)	0
**Uninvolved**	128 (97.7)	114 (97.4)	14 (100)
**Complications,** ***N*** **(%)**			
**Hematoma**	1 (0.7)	1 (0.8)	0
**Delayed wound healing**	0	0	0
**Seroma requiring needle aspiration**	9 (6.9)	8 (6.8)	1 (6.7)
**Small blister formation**	0	0	0
**Transient NAC ischemia**	7 (5.3)	7 (6)	–
**Partial NAC necrosis**	7 (5.3)	7 (6)	–
**Total NAC necrosis**	1 (0.7)	1 (0.8)	–
**Temporal color change of skin flap from decreased blood flow**^**a**^	4 (3.0)	4 (3.4)	0
**Partial/Full thickness skin flap necrosis**	0	0	0
**Implant Infection**	2 (1.5)	2 (1.7)	0
**Implant loss**	1 (0.7)	1 (0.8)	0
**Surgical complications by Clavien-Dindo classification,** ***N*** **(%)**			
I	28 (21.5)	27 (23.1)	1 (6.7)
**II**	1 (0.7)	1 (0.8)	0
**IIIa**	1 (0.7)	1 (0.8)	0
**IIIb**	2 (1.5)	2 (1.7)	0
**IVa**	0	0	0
**IVb**	0	0	0
**V**	0	0	0
**Oncologic safety,** *N* **(%)**			
**Locoregional recurrence (yes)**	2 (1.5)	2 (1.7)	0
**Distant metastasis (yes)**	9 (6.9)	9 (7.7)	0
**Mortality (yes)**	2 (1.5)	2 (1.7)	0
**Follow-up time, months, mean ± SD (median, range)**	34.5 ± 15.6 (36, 10–60)	36.7 ± 14.5 (37, 10–60)	13.6 ± 8.8 (11, 10–4)

*S-P 3D E-NSM* single-port 3-dimensional endoscopic-assisted nipple-sparing mastectomy, *S-P 3D E-BCS* single-port 3-dimensional endoscopic-assisted breast-conserving surgery, *LD* latissimus dorsi myocutaneous flap, *NAC* nipple-areolar complex, *SD* standard deviation

^a^All patients facing skin flap ischemia had temporal color change on 1–10% of breast surface area, which were reversible without the need of antibiotics or surgical procedure

For the treatment of gynecomastia in male patients, 10 cases of S-P 3D E-subcutaneous mastectomy were performed. The mean age of the patients was 27.2 ± 6.7 years, 80% (8/10) were BMI > 24 kg/m^2^, and 20% (2/10) were BMI 18–24 kg/m^2^. According to Simon classification [27], 20% (2/10) of patients had severity grade I (small enlargement without skin excess), 70% (7/10) of them had severity grade IIa (moderate enlargement without skin excess), and the remaining 10% (1/10) of patients had severity grade IIb (moderate enlargement with minor skin excess). Regarding perioperative outcomes in this patient group, the mean total operative time was 310 ± 70 min, mean blood loss 57.5 ± 35.8 ml, mean specimen weight 436.4 ± 261 g, and length of hospital stay 1.8 ± 0.6 days. Clinical outcomes of gynecomastia patients receiving S-P 3D E-subcutaneous mastectomy were shown in Table 3.

**Table 3:** Clinical outcomes of gynecomastia patients who received single-port 3-dimensional (3D) endoscopic-assisted subcutaneous mastectomy

**Gynecomastia patients (*N* = 10)**	
**Age, years, *N* ( %)**	
** < 40**	9 (90)
**≧ 40, < 60**	1 (10)
**≧ 60**	0
**Mean ± SD**	27.2 ± 6.7
**BMI, kg/m**^**2**^**, *N* (%)**	
** < 18**	0
**18–24**	2 (20)
** > 24**	8 (80)
**Grade of gynecomastia**^**a**^**, *N* (%)**	
**Grade I (small enlargement without skin excess)**	2 (20)
**Grade IIa (moderate enlargement without skin excess)**	7 (70)
**Grade IIb (moderate enlargement with minor skin excess)**	1 (10)
**Grade III (marked enlargement with excess skin and ptosis)**	0
**Perioperative parameters for bilateral subcutaneous mastectomy**	**Mean ± SD (median, range)**
**Total operation time, min**	310 ± 70 (312.5, 205–440)
**Blood loss, ml**	57.5 ± 35.8 (50, 30–150)
**Mastectomy specimen weight, g**	436.4 ± 261 (445.25, 140–931)
**Length of hospital stay, days**	1.8 ± 0.6 (2, 1–3)
**Follow-up durations, months, mean ± SD (median, range)**	3.3 ± 4.2 (1.5, 0–14)

*BMI* body mass index, *SD* standard deviation

^a^Classified by Simon et al. in Simon BE, Hoffman S, Kahn S. Classification and surgical correction of gynecomastia. Plast Reconstr Surg 1973;51:48–52

### Patient-reported aesthetic results

83.8% (98/117) of breast cancer patients who received treatment by S-P 3D E-NSM answered the questionnaire. Regarding the results from 68 patients receiving S-P 3D E-NSM with immediate breast reconstruction (Table 4), 83.8% of them were satisfied with cosmetic outcomes while wearing clothes, 16.2% reported “fair,” and none of them reported poor results. 69.7% of the patients reported “good and excellent” breast symmetry, while 25.8% reported “fair” outcome. 79.1% of them were satisfied with the NAC position, 20.9% showed fair results, and none reported poor NAC location. However, 2 patients (2.9%) reported poor satisfaction without clothes, and 4 patients (6%) showed poor satisfaction with bilateral breast size and symmetry. The overall score results demonstrated “excellent” in 35.3% (24/68), “good” in 48.5% (33/68), and “fair” in 16.2% (11/68) of patients. None of the patients reported poor overall aesthetic outcomes. 80.9% of the patients reported that they would choose the same operation again if given the chance to do so.

**Table 4:** Patient-reported cosmetic outcomes after receiving S-P 3D E-NSM with immediate breast reconstruction

Questions (*N* = 68), *N* (%)	Poor	Fair	Good	Excellent	Mean score
**Q1: preoperative breast appearance satisfaction**	1 (1.5)	17 (25)	34 (50)	16 (23.5)	2.9 ± 0.7
**Q2: postoperative breast appearance satisfaction—with clothes**	0	11 (16.2)	31 (45.6)	26 (38.2)	3.2 ± 0.7
**Q3: postoperative breast appearance satisfaction—without clothes**	2 (2.9)	15 (22.1)	35 (51.5)	16 (23.5)	2.9 ± 0.8
**Q4: postoperative bilateral breast size satisfaction (NA = 1)**	1 (1.5)	19 (28.3)	30 (44.8)	17 (25.4)	2.9 ± 0.8
**Q5: postoperative bilateral breast symmetry satisfaction (NA = 2)**	3 (4.5)	17 (25.8)	28 (42.4)	18 (27.3)	2.9 ± 0.8
**Q6: postoperative nipple-areola position satisfaction (NA = 1)**	0	14 (20.9)	30 (44.8)	23 (34.3)	3.1 ± 0.7
**Q7: scar appearance satisfaction (NA = 2)**	0	8 (12.1)	24 (36.4)	34 (51.5)	3.4 ± 0.7
**Q8: scar length satisfaction (NA = 1)**	0	9 (13.5)	22 (32.8)	36 (53.7)	3.4 ± 0.7
**Q9: surgical wound position satisfaction (NA = 1)**	1 (1.5)	6 (8.9)	20 (29.9)	40 (59.7)	3.5 ± 0.7
**Q10: are you willing to undergo S-P 3D E-NSM with immediate reconstruction again if you could choose?**	**Yes**		**No**	**Not sure**	
	55 (80.9)		9 (13.2)	4 (5.9)	
**Overall score**	**Poor (8–11)**	**Fair (12–19)**	**Good (20–27)**	**Excellent (28–32)**	
*N* (%)	0	11 (16.2)	33 (48.5)	24 (35.3)	

This questionnaire consisted of 10 questions and 4 itemized scales graded. Evaluate the overall satisfaction score of questions 2 to 9 for each patient. The aesthetic results indicated by the overall scores were as follows: 8–11 (poor), 12–19 (fair), 20–27 (good), and 28–32 (excellent). Patients with aesthetic results as “excellent” or “good” are defined as satisfied with the cosmetic outcome

*S-P 3D E-NSM* single-port 3-dimensional endoscopic-assisted nipple-sparing mastectomy

According to 30 patients who undergone S-P 3D E-NSM without reconstruction (Additional file 1: Table S1), 66.7% of patients reported “good” and “excellent,” and the remaining 33.3% reported “fair” to the overall cosmetic satisfaction. None of them reported “poor” overall aesthetic satisfaction, regardless of the skin reduction procedure. 53.3% and 61.9% of them were satisfied with cosmetic outcomes without clothes and bilateral breast size, respectively. Ninety percent of the patients reported that they would choose the same operation again if given the chance to do so.

## Discussion

In the current study, we aimed to demonstrate perioperative outcomes and patient-reported aesthetic satisfaction with our innovative S-P 3D EABS. We conducted a retrospective analysis of 145 patients who underwent a total of 164 breast operations, which were 131 patients with breast cancer (Figs. 2 and 3) including 13 patients receiving prophylactic mastectomy and 10 male patients with bilateral gynecomastia (Fig. 4). Our findings demonstrated that this technique yielded acceptable complications and satisfactory aesthetic results.

The introduction of S-P 3D EABS in breast surgery represents a significant advancement in minimal-accessed breast surgery [3, 10, 11, 29]. At our center, we initiated the use of S-P 3D E-NSM in August 2018 [11] by addressing challenges faced during previous dual-incision 2D endoscopic surgery with retractors [1, 2, 14, 15, 30–32]. Our approach involved performing endoscopic resection through a single, inconspicuous axillary incision, which aimed to optimize cosmetic outcomes while preserving blood supply to the NAC and skin flap [3]. Additionally, to ensure proper exposure from a distant incision, we replaced manual retraction with an air-insufflation system, which could minimize the burdensome for surgical assistants and reduce risks of skin flap or NAC ischemia/necrosis [3]. Furthermore, the integration of 3D videoscope technology offered improvement in depth perception and clear visualization through high-resolution imaging [12, 13]. These advancements have resulted in more precise surgical resection, comparable to the benefits seen in robotic breast surgery but at a significantly lower cost [29]. This aspect is particularly relevant for patients in low- to middle-income countries, where cost considerations are crucial.

In our center, the most common immediate reconstruction after S-P 3D E-NSM is direct-to-implant breast reconstruction in the subpectoral plane (Table 1). This approach involves utilizing a single axillary incision, which serves multiple purposes, including harvesting the sentinel lymph node (SLN), performing the S-P 3D E-NSM procedure, removing the specimen, and creating a subpectoral pocket for reconstruction. However, in cases where patients have a large specimen, an extension of the axillary incision is made longitudinally at the lateral part of the chest (intercostal space 3rd–6th, mid-axillary line) to facilitate the surgical exposure during mastectomy or ALND, specimen removal, and provide an option for additional volume replacement reconstruction, such as autologous pedicle TRAM flap (Fig. 2).

In our study, none of the patients required conversion to open surgery (Table 2). The use of 3D EABS resulted in less intraoperative blood loss, which can be attributed to the positive pressure of the air inflation system and clear visualization provided by the 3D imaging system, which facilitates precise resection and decreases a risk of bleeding from perforators [3, 33–36]. The operative time of S-P 3D E-NSM may appear longer than previous studies and conventional breast surgery [33–35, 37–40]; however, our preliminary cumulative sum (CUSUM) plot analysis from 80 cases of S-P 3D E-NSM and ipsilateral gel implant-based reconstruction [3, 24] demonstrated that intraoperative blood loss and operative time significantly decrease after the learning curve of surgeons which required 27 cases to be overcome. Our length of hospital stay was comparable to previous studies [33, 37, 39, 41].

In terms of S-P 3D E-BCS, it seems to have a longer operative time, but offers better cosmetic outcomes compared to conventional BCS in previous studies [42–48]. However, there is a lack of a large study comparing endoscopic and conventional BCS. Therefore, we are conducting a study comparing conventional and endoscopic breast surgery in our center.

Regarding perioperative complications, we found 32 events reported, which were mostly classified in CD classification I–II [22, 49](Table 2). Our results exhibited a lower incidence of NAC and skin flap ischemia/necrosis compared to previous studies [33, 37, 40]. This can be attributed to our single incision at an axilla or lateral chest, which did not disrupt blood flow to the NAC or skin flap over the breast [3, 10, 24, 25, 29, 50]. Additionally, the utilization of an air inflation system provided an adequate surgical exposure while minimizing traumatized traction force on the skin flap. Our hydro-dissection technique using a tumescent solution containing adrenaline might result in reduced blood loss, thereby decreasing thermal burns from excessive electrocauterization.

Clear visualization from the 3D imaging system also helped in identifying and preserving perforators that supplied blood flow to the NAC and skin flap [3, 11, 29, 49]. As a result, all these factors may contribute to the lower rate of NAC and skin flap ischemia. Furthermore, 95% (18/19) of NAC/flap ischemic events were reversible and successfully treated with local wound care at the outpatient clinic (CD I) [22]. Complications categorized as CD classification III [22] were reported in only 3 (2.2%) patients. One patient had a total NAC necrosis requiring a local debridement (CD IIIa). The remaining 2 patients were classified as CD IIIb, with one suffering from an implant infection requiring surgical drainage under general anesthesia, and the other with an implant infection requiring an explantation. None of the patients had been reported in CD classification IV or V.

Margin involvement is one of the major challenge in EABS for breast cancer patients [33, 36]. However, our results demonstrated an acceptable incidence of involved margins compared to previous studies [33, 51]. None of the patients receiving S-P 3D E-BCS in our study had an involved margin in their pathological reports, while only three patients (2.6%) who underwent S-P 3D E-NSM showed an involved margin (Table 2). This outcome may be attributed to our precise determination of resection margins using intraoperative ultrasonography and the utilization of breast specimen mammography to confirm adequate resection for S-P 3D E-BCS.

During the mean follow-up time of 34 months, we observed local recurrence in 2 patients (1.5%) and distant metastasis in nine patients (6.9%), with two of them suffering from multiple metastases after the surgery reported mortality (1.5%) from disease progression. Our incidences of short-term oncological outcomes were comparable to previous studies [6, 7, 23, 30, 33, 34, 36, 47, 52–55].

Our concept of S-P 3D EABS can also be applied for the treatment of gynecomastia in male patients (Fig. 4). Compared with a periareolar incision in conventional subcutaneous mastectomy, a longitudinal incision at the lateral chest provides a less noticeable surgical scar and potentially preserves the blood flow of the NAC and skin flap (Table 3). Therefore, S-P 3D E-subcutaneous mastectomy could be a promising surgical alternative in this group of patients [27].

In accessing patient-reported cosmetic satisfaction (Table 4), 98 (83.8%) patients who underwent S-P 3D E-NSM responded to our questionnaire, including 18 from 24 patients who experienced postoperative complications. The majority of patients who undergone S-P 3D E-NSM expressed satisfaction with their breast appearance (75%) and scar appearance (87.9%), breast symmetry (69.7%), and NAC position (79.1%). The overall satisfaction was reported as “excellent” in 35.3% of patients and “good” in 48.5% of them. Furthermore, 80.9% of patients would choose S-P 3D E-NSM with immediate reconstruction again if given the opportunity. However, there were 6 patients who reported poor satisfaction with cosmetic appearance without clothes, bilateral breast size, or bilateral symmetry. This may be due to most of our breast reconstruction being gel implant which was possible to have bilateral asymmetry in some patients who had very small breasts. As for the patients receiving S-P 3D E-NSM without reconstruction, we have observed skin retraction gradually after the removal of breast tissue leaving a breast-like contour of the chest wall (Fig. 2(2.5)), and the result from our questionnaire showed that 66.7% of patients reported “good” and “excellent” and the remaining 33.3% reported “fair” to the overall cosmetic satisfaction, even none of the skin reduction procedure was performed (Additional file 1: Table S1).

Patient selection is one of the important factors to the success of EABS. We consider EABS in early-stage breast cancer and no severe medical comorbidity as mentioned before. Regarding the patient’s breast size and shape, the ideal candidates for S-P 3D E-NSM were patients with small to medium-sized breasts (≤ cup C or approximately < 450 g of breast specimen weight) and no/mild ptosis [3, 11]. However, when evaluating candidates for S-P 3D E-BCS, we consider the correlation between breast tumor size and the tumor location. For example, patients with medium-sized breasts (cup B or C) who have small tumor sizes (less than 2 cm) located at the upper outer, upper inner, or lower outer quadrants are preferable as they provide adequate space for dissection and a single port placement. However, a tumor at the lower-inner quadrant is not contraindicated for S-P 3D E-BCS, and an additional peri-areolar incision should be considered to facilitate an endoscopic resection. From our experiences, 2 patients with a tumor in the lower-inner area (one of them could undergo S-P 3D E-BCS through a single axillary incision and another was performed with dual axillary and peri-areolar incisions) reported no complication and comparable hospital stay.

One of the technical challenges encountered in our S-P 3D EABS with air insufflation is the evacuation of smoke generated during the surgery. To solve this issue, our approach involves connecting the endoscopic working instruments to Nelaton tubes, which are then attached to a suction device (Fig. 1j). This setup enables the aspiration of smoke in the surgical field, ensuring clear visibility and maintaining a safe operating environment.

While our study provides valuable insights, it is important to acknowledge certain limitations. The retrospective design of this study introduces the possibility of confounding factors that may influence the results. First, it could contain a recall bias in cosmetic outcome measurement, which some participants were not willing to answer the questionnaire in the outpatient clinic, and our questionnaire still lacks a formal validation. Furthermore, the follow-up period in our study is currently limited, especially those of S-P 3D E-BCS that was established in 2022. However, we keep monitoring the patients for longer follow-up time to achieve a comprehensive evaluation of the oncological safety of this technique. Another aspect that should be noted is the relatively small sample size and lack of direct comparison between 3D EABS and conventional, 2D EABS, or robotic breast surgery in our study. However, it is worth mentioning that this technique is increasingly being adopted in our institute, and we are collecting additional data to conduct further investigations on the efficacy and outcomes of S-P 3D EABS. By expanding our dataset, we aim to enhance our understanding of this technique and its potential benefits. Despite these limitations, our study provides a foundation for future research and highlights the improvement in the field of EABS.

## Conclusion

Single-port 3D EABS is a promising minimal-accessed breast surgery technique, demonstrating its safety and efficacy in the treatment of both malignant and benign breast conditions, while also offering remarkable patient-reported aesthetic satisfaction.

### Supplementary Information

**Additional file 1: Table S1.** Patient-reported cosmetic outcomes after receiving S-P 3D E-NSM* without reconstruction. This questionnaire consisted of 10 questions and 4 itemized scales graded. Evaluate the overall satisfaction score of questions 2 to 9 for each patient. The aesthetic results indicated by the overall scores were as follows: 8- 11(poor), 12–19 (fair), 20 -27(good), and 28- 32(excellent). Patients with aesthetic results as “excellent” or “good” are defined as satisfied with the cosmetic outcome. *S-P 3D E-NSM = single-port 3-dimensional endoscopic-assisted nipple-sparing mastectomy.

## Data Availability

The datasets used and/or analyzed during the current study are available from the corresponding author (Hung-Wen Lai) upon reasonable request.

## Abbreviations

S-P 3D EABS: Single-port 3-dimensional endoscopic-assisted breast surgery
S-P 3D E-NSM: Single-port 3-dimensional endoscopic-assisted nipple-sparing mastectomy
S-P 3D E-BCS: Single-port 3-dimensional endoscopic-assisted breast-conserving surgery
BMI: Body mass index
ALN: Axillary lymph node
SLNB: Sentinel lymph node biopsy
ALND: Axillary lymph node dissection
TRAM flap: Transverse rectus abdominis myocutaneous flap
LD flap: Latissimus dorsi myocutaneous flap
