# Supplementary material for: Single-port three-dimensional (3D) endoscopic-assisted breast surgery—preliminary results and patient-reported satisfaction in 145 breast cancer and gynecomastia cases
Source: World J Surg Oncol. 2023 Oct 26;21:335. doi: 10.1186/s12957-023-03191-7 (PMC10601236; doi:10.1186/s12957-023-03191-7)
Supplement: Supplementary file 1 — Additional file 1: Table S1. Patient-reported cosmetic outcomes after receiving S-P 3D E-NSM* without reconstruction. This questionnaire consisted of 10 questions and 4 itemized scales graded. Evaluate the overall satisfaction score of questions 2 to 9 for each patient. The aesthetic results indicated by the overall scores were as follows: 8- 11(poor), 12–19 (fair), 20 -27(good), and 28- 32(excellent). Patients with aesthetic results as “excellent” or “good” are defined as satisfied with the cosmetic outcome. *S-P 3D E-NSM = single-port 3-dimensional endoscopic-assisted nipple-sparing mastectomy. [file 12957_2023_3191_MOESM1_ESM.docx]

**Supplementary table 1** Patient-reported cosmetic outcomes after receiving S-P 3D E-NSM* without reconstruction

| Questions (N = 30), N(%) | Poor | Fair | Good | Excellent | Mean score |
| --- | --- | --- | --- | --- | --- |
| Q1: preoperative breast appearance satisfaction | 1(3.3) | 6(20) | 18(60) | 5(16.7) | 2.9 ± 0.7 |
| Q2: postoperative breast appearance satisfaction–with clothes | 0 | 14(46.7) | 12(40) | 4(13.3) | 2.7 ± 0.7 |
| Q3: postoperative breast appearance satisfaction–without clothes | 3(10) | 11(36.7) | 13(43.3) | 3(10) | 2.5 ± 0.8 |
| Q4: postoperative bilateral breast size satisfaction (NA = 9) | 0 | 8(38.1) | 11(52.4) | 2(9.5) | 2.7 ± 0.6 |
| Q5: postoperative bilateral breast symmetry satisfaction (NA = 10) | 0 | 7(35) | 10(50) | 3(15) | 2.8 ± 0.7 |
| Q6: postoperative nipple-areola position satisfaction (NA = 1) | 0 | 10(34.5) | 14(48.3) | 5(17.2) | 2.8 ± 0.7 |
| Q7: scar appearance satisfaction | 1(3.3) | 7(23.3) | 14(46.7) | 8(26.7) | 3 ± 0.8 |
| Q8: scar length satisfaction | 0 | 7(23.3) | 16(53.4) | 7(23.3) | 3 ± 0.7 |
| Q9: surgical wound position satisfaction | 0 | 7(23.3) | 16(53.4) | 7(23.3) | 3 ± 0.7 |
| Q10: are you willing to undergo single-port 3D E-NSM again if you could choose? (NA = 10) | **Yes** | | | **No** | |
|  | 18(90) | | | 2(10) | |
| Overall score: | **Poor**  **(8-11)** | **Fair**  **(12-19)** | **Good**  **(20-27)** | **Excellent (28-32)** |  |
| N(%) | 0 | 10(33.3) | 14(46.7) | 6(20) |  |

This questionnaire consisted of 10 questions and 4 itemized scales graded. Evaluate the overall satisfaction score of questions 2 to 9 for each patient. The aesthetic results indicated by the overall scores were as follows: 8- 11(poor), 12-19 (fair), 20 -27(good), and 28- 32(excellent). Patients with aesthetic results as ‘‘excellent’’ or ‘‘good’’ are defined as satisfied with the cosmetic outcome.

* S-P 3D E-NSM = single-port 3-dimensional endoscopic-assisted nipple-sparing mastectomy
